# The impact of multidisciplinary pre-dialysis care on the outcomes of incident peritoneal dialysis patients

**DOI:** 10.1186/s12882-022-02800-z

**Published:** 2022-05-05

**Authors:** I-Kuan Wang, Tung-Min Yu, Tzung-Hai Yen, Hei-Tung Yip, Ping-Chin Lai, Chi-Yuan Li, Kuo-Ting Sun, Fung-Chang Sung

**Affiliations:** 1 Graduate Institute of Biological Sciences, College of Medicine, China Medical University, Taichung, Taiwan; 2 Divisions of Nephrology, China Medical University Hospital, Taichung, Taiwan; 3 Department of Medicine, College of Medicine, China Medical University, Taichung, Taiwan; 4 Division of Nephrology, Taichung Veterans General Hospital, Taichung, Taiwan; 5 Division of Nephrology, Chang Gung Memorial Hospital, Taipei, Taiwan; 6 Chang Gung University College of Medicine, Taoyuan, Taiwan; 7 Management Office for Health Data, China Medical University Hospital, Taichung, Taiwan; 8 Department of Anesthesiology, China Medical University Hospital, Taichung, Taiwan; 9 Department of Dentistry, China Medical University Hospital, Taichung, Taiwan; 10 Department of Health Services Administration, China Medical University College of Public Health, Taichung, 404 Taiwan; 11 Department of Food Nutrition and Health Biotechnology, Asia University, 500 Lioufeng Rd., Wufeng, Taichung, 413 Taiwan

**Keywords:** Multidisciplinary pre-dialysis care, End-stage kidney disease, Patient survival, Peritoneal dialysis, Peritonitis, Technique survival

## Abstract

**Background:**

This study aims to evaluate the impact of multidisciplinary pre-dialysis care (MDPC) on the risks of peritonitis, technique failure and mortality in peritoneal dialysis (PD) patients.

**Methods:**

Incident end-stage kidney disease patients who received peritoneal dialysis (PD) for more than 90 days were recruited in this study from 1 January 1, 2007 to December 31, 2018. Patients were classified into two groups, the MDPC group and the control group, that received the usual care by nephrologists. Risks of the first episode of peritonitis, technique failure and mortality were compared between the two groups.

**Results:**

There were 126 patients under the usual care and 546 patients under the MDPC. Patients in the MDPC group initiated dialysis earlier than those in the non-MDPC group. There was no significant difference between these two groups in time to the first episode of peritonitis. Compared to the non-MDPC group, the MDPC group was at similar risks of technique failure (adjusted HR = 0.85, 95% CI = 0.64–1.15) and mortality (adjusted HR = 0.66, 95% CI = 0.42–1.02). Among patients with diabetes, the risk of mortality was significantly reduced in the MDPC group with an adjusted HR of 0.45 (95% CI = 0.25–0.80).

**Conclusions:**

There was no significant difference in time to develop the first episode of peritonitis, and risks of technique failure and mortality between these two groups. Diabetic PD patients under MDPC had a lower risk of mortality than those under the usual care.

## Introduction

Chronic kidney disease (CKD), defined by reduced glomerular filtration rate, proteinuria or structural kidney disease, is an important global health issue with the prevalence around 12–16% and high associated mortality [1, 2]. CKD is generally progressive and irreversible, and could progress to end-stage kidney disease (ESKD). Patients with ESKD require renal replacement therapy to maintain their lives. Because of a shortage of organ donation, the majority of ESKD patients in Taiwan undergo maintenance dialysis, including hemodialysis (HD) and peritoneal dialysis (PD) [3]. PD is cost-effective dialysis modality associated with better preservation of residual renal function compared to HD [4, 5]. However, the majority of ESKD patients in Taiwan received HD rather than PD. A higher technique failure rate and a higher risk of peritonitis in patients treated with PD compared to those treated with HD are major challenges for caring PD patients [6, 7].

As patients with CKD usually have multiple coexisting comorbidities, a coordinated multidisciplinary care may be needed to improve the management and outcome of these patients [8–14]. A nationwide multidisciplinary pre-dialysis care (MDPC) program has been established since November 2006 in Taiwan to improve the quality and outcomes of pre-dialysis care. The team of MDPC consists of nephrologists, dietitians and nurses and provides standardized pre-dialysis education according to the Kidney Disease Outcomes Quality Initiative guidelines. Dietitians provide dietary consultation. Nurses of case management contact patients to ensure regular follow-up and deliver knowledge of nutrition, life style modification, nephrotoxin avoidance, medication, risk factors and complications of kidney disease, pre-dialysis preparation, and dialysis modality every 1–3 months. The benefits, disadvantages and self-care for different renal replacement modalities are explained. However, studies about the impact of MDPC on the outcome of PD patients are limited [13, 15]. The aim of this study is to evaluate the impact of MDPC on the risks of post-dialysis peritonitis, technique failure and mortality in PD patients using recent data at a tertiary medical center in Taiwan.

## Materials and methods

### Data source

The medical records of patients with ESKD undergoing PD from January 1, 2007 to December 31, 2018 were collected for this study at the China Medical University Hospital, one of the major teaching medical centres in Taiwan. The medical records contained the information of demographic data, medical history, underlying comorbid conditions, laboratory data and treatment at the beginning of the PD therapy. This study was performed in compliance with guidelines of the Declaration of Helsinki. This retrospective observational study was approved by the Research Ethics Committee of China Medical University Hospital [CMUH103-REC2-070 (CR5)]. Because this study involved retrospective review of existing data, the Research Ethics Committee of China Medical University Hospital specifically waived the need for informed consent.

### Study population

Patients aged 18 years and older receiving PD for more than 90 days were identified and classified into two groups: those who had received the MDPC program as the study group and those who received the usual care by nephrologists as the control group. The pre-dialysis was defined as at least 90 days before the initiation of dialysis. An earlier study from our center revealed that icodextrin use was associated with lower risks of both technique and death [16]. A recent study also from our center demonstrated that APD is associated a lower risk of technique failure than CAPD [17]. Thus, the use of icodextrin or APD was considered as a covariate in data analysis. Patients who had received icodextrin for at least 30 days were defined as the users of icodextrin. Similarly, patients who received APD for at least 30 days were defined as the users of APD. The others were classified as the continuous ambulatory peritoneal dialysis (CAPD) group. The dose of PD was prescribed incrementally to aim for a weekly Kt/V ≥ 1.7 [18, 19]. The adequacy of PD was measured one month after the initiation of PD and every 6 months. All patients were followed up until transfer from PD to HD, renal transplantation, transfer to another hospital, death, or December 31, 2018, whichever came first.

### Outcomes and covariates

During the follow-up period, incident rates of the first episode of peritonitis, technique failure, and mortality were estimated. Demographic variables included age, gender, life style variables of smoking and alcohol drinking, comorbidities, laboratory data, and treatment.

### Statistical analysis

The baseline characteristics between patients with and without MDPC were compared and tested by Chi-square test and Student’s t test for categorical variables and continuous variables, respectively. The Kaplan–Meier method was used to estimate and plot cumulative incidence of outcomes. The multivariate Cox proportional hazards model was used to estimate the adjusted hazard ratio (HR) and 95% CI after controlling for variables with a p value < 0.25 in the univariate Cox model. The subhazard ratio (SHR) and 95% confidence interval (CI) was also calculated with considering deaths as a competing risk [20]. Technique failure was defined as transfer to HD for at least 30 days or death on PD [21, 22]. Death is one of the major causes of drop-out in PD patients. In addition, complications of PD such as ultrafiltration failure, peritonitis, mineral bone disease, cardiovascular disease, and encapsulating peritoneal sclerosis may lead to death. Thus, death was included as a cause of technique failure. Renal transplantation, transfer to another hospital for care, and alive at the end of the study period were censored for technique survival analysis. If patients died within 90 days after switching to HD, the death was attributed to PD and counted as a death event. Otherwise, transfer to HD, renal transplantation, transfer to other hospital for care, and alive at the end of the study period (December 31, 2018) were censored for patient survival analysis. Stratification analysis by diabetes status was also performed to estimate its impact on outcomes. The statistical software SAS (version 9.4; SAS Institute, Inc., Cary, NC, USA) and R (version 2.1) was utilized to perform the analysis.

## Results

There were 126 patients under the usual care and 546 patients under the MDPC program. 34 patients were transferred to other hospitals and 43 patients received transplantation in the MDPC group. 6 patients were transferred to other hospitals and 8 patients underwent transplantation in the control group. The mean follow-up time for patients in MDPC and usual care groups were 5.20 ± 3.18 years and 5.41 ± 3.48 years (*p* = 0.53), respectively. The MDPC group consisted of more women and elderly patients than the control group (Table 1). Less than 20% of patients were smokers or had alcohol drinking. Patients in the MDPC group were more likely to use automated peritoneal dialysis (APD) and less likely to have gout. 3 patients and 5 patients in the MDPC and control groups had anuria respectively (data not shown). The MDPC group had a higher mean renal Kt/V than that in control patients (0.66 ± 0.43 versus (vs.) 0.47 ± 0.36, *p* < 0.001), but a lower mean peritoneal Kt/V (1.31 ± 0.36 vs 1.46 ± 0.37, *p* < 0.001). However, there was no difference in renal Kt/V between the two group for diabetes patients (1.90 ± 0.38 versus (vs.) 1.89 ± 0.41, *p* = 0.90). The proportion of patients under MDPC increased in the most recent year. The top three causes of ESKD were diabetes, chronic glomerulonephritis and hypertension.

**Table 1:** Baseline characteristic of patients with and without multidisciplinary pre-dialysis care

	Multidisciplinary care				
	No		Yes		
	*N* = 126		*N* = 546		
Variables	n	%	n	%	*p*-value
Gender					0.65
Female	63	50%	288	53%	
Male	63	50%	258	47%	
Age, years					0.005
18–30	9	7%	15	3%	
31–50	40	32%	166	30%	
50–70	66	52%	258	47%	
> 71	11	9%	107	20%	
mean, (SD)	52.2	(13.7)	56.5	(14.4)	0.002
Smoking					0.09
Current	14	13%	47	9%	
Ever	8	7%	22	4%	
Alcohol drinking					0.26
Current	2	2%	8	1%	
Ever	7	7%	18	3%	
Comorbidities					
Diabetes	61	48%	236	43%	0.34
Hypertension	93	74%	415	76%	0.39
Cardiovascular disease	30	24%	139	25%	0.79
Liver cirrhosis	1	1%	18	3%	0.22
Gout	14	11%	31	6%	0.05
Cancer	2	2%	16	3%	0.59
TB	0	0%	1	0.2%	-
HBV	20	16%	59	11%	0.15
HCV	9	7.1%	39	7.1%	1.00
Icodextrin use	54	43%	190	35%	0.11
APD use	28	22%	219	40%	< 0.001
PET					0.72
Low/low average	44	35%	183	34%	
High/high average	79	63%	362	66%	
Systolic blood pressure (mmHg)					0.76
mean, (SD)	142.7	(24.4)	143.4	(21.8)	
Diastolic blood pressure (mmHg)					1.00
mean, (SD)	81.3	(13.7)	81.3	(14.4)	
Kt/V					0.32
mean, (SD)	1.93	(0.38)	1.97	(0.43)	
Renal, Kt/V					< 0.001
mean, (SD)	0.47	(0.36)	0.66	(0.43)	
Peritoneal, Kt/V					< 0.001
mean, (SD)	1.46	(0.37)	1.31	(0.36)	
Albumin (g/dL)					0.42
mean, (SD)	3.53	(0.47)	3.57	(0.51)	
nPNA (g/kg/day)					0.50
mean, (SD)	1.04	(0.24)	1.05	(0.26)	
P (mg/dL)					0.08
< 3.5	8	6%	30	5%	
3.5–5.5	58	46%	230	42%	
> 5.5	60	48%	286	52%	
mean, (SD)	5.45	(1.52)	5.73	(1.62)	0.07
Hb (g/dL)					0.75
mean, (SD)	9.98	(1.37)	9.93	(1.44)	
HbA1c					0.64
mean, (SD)	6.75	(1.34)	6.83	(1.47)	
Years of dialysis initiation					0.04
2007–2010	39	31%	137	25%	
2011–2014	62	49%	240	44%	
2015–2017	25	20%	169	31%	
Break in period, days					
mean, (SD)	20.9	(77.7)	25.9	(65.3)	0.51
Etiology of ESKD					0.46
Diabetes	55	44%	215	39%	
Chronic glomerulonephritis	45	36%	202	37%	
Hypertension	12	10%	66	12%	
Chronic tubulointerstital disease	11	9%	32	6%	
Adult polycystic kidney disease	1	1%	14	3%	
Obstructive uropathy	0	0%	8	1%	
Others	2	2%	9	2%	

*SD* Standard deviation, *TB* Tuberculosis, *HBV* Hepatitis B virus, *HCV* Hepatitis C virus, *APD* Automated peritoneal dialysis, *PET* Peritoneal equilibrium test, *nPNA* Normalized protein nitrogen appearance, *Hb* Haemoglobin, *HbA1c* Glycated haemoglobin

**Fig. 1:**
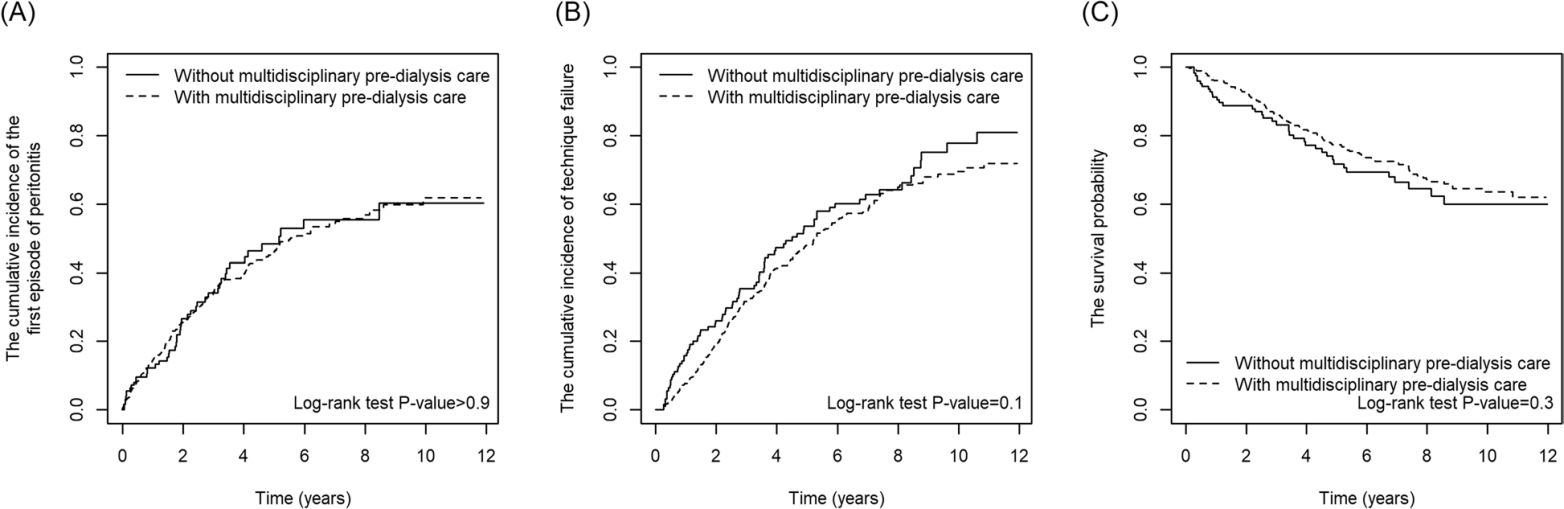
The cumulative incidence of the first episode of peritonitis (**A**), technique failure (**B**) and survival probability (**C**) in patients with and without multidisciplinary pre-dialysis care

Figure 1 shows that the cumulative incident rates of the first episode of peritonitis, and technique failure and survival probability of patients were not different between the MDPC and control groups.

The incident rates of the first episode of peritonitis were similar between the MDPC group and controls (Table 2). The incidence of technique failure was lower in the MDPC group than in controls, but the estimated HRs were all not significant. The MDPC group had a lower mortality rate than the control group (0.47 versus 0.56 per 10 person-years), with an adjusted HR of 0.63 (95% CI = 0.41–0.97) after controlling for gender, age, smoking, diabetes, hypertension, cardiovascular disease, liver cirrhosis, gout, hepatitis C, icodextrin use, APD use, peritoneal permeability, Kt/V, albumin and hemoglobin. Using another model replacing total Kt/V by renal Kt/V, the adjusted HR for mortality became 0.66 (95% CI = 0.42–1.02).

**Table 2:** The risk of the first episode of peritonitis, technique failure and mortality in patients with and without multidisciplinary pre-dialysis care

	Multidisciplinary pre-dialysis care		
Event	No (*N* = 126)	Yes (*N* = 546)	*p*-value
The first episode of peritonitis			
n	47	211	
Person-years	360	1616	
Incidence rate^a^	1.30	1.31	
cHR (95% CI)	1.00 (reference)	1.00 (0.73,1.37)	0.98
aHR^b^ (95% CI)	1.00 (reference)	1.15 (0.83,1.61)	0.41
aHR^c^ (95% CI)	1.00 (reference)	1.19 (0.85,1.67)	0.31
aSHR^b^ (95% CI)	1.00 (reference)	1.15 (0.83,1.61)	0.40
aSHR^c^ (95% CI)	1.00 (reference)	1.20 (0.85,1.67)	0.30
Technique failure			
n	76	276	
Person-years	518	2258	
Incidence rate^a^	1.47	1.22	
cHR (95% CI)	1.00 (reference)	0.83 (0.64,1.07)	0.14
aHR^d^ (95% CI)	1.00 (reference)	0.86 (0.64,1.15)	0.31
aHR^e^ (95% CI)	1.00 (reference)	0.85 (0.64,1.15)	0.29
Mortality			
n	38	113	
Person-years	681	2837	
Incidence rate^a^	0.56	0.47	
cHR (95% CI)	1.00 (reference)	0.83 (0.58,1.19)	0.30
aHR^f^ (95% CI)	1.00 (reference)	0.63 (0.41,0.97)	0.04
aHR^g^ (95% CI)	1.00 (reference)	0.66 (0.42,1.02)	0.06

*cHR* Crude hazard ratio, *aHR* Adjusted hazard ratio, aSHR Adjusted sub-distribution hazard ratio

^a^incidence rate per 10 person-years

^b^adjusted for gender, diabetes, hypertension, HBV, HCV, icodextrin use, APD use, Kt/V, albumin and years of dialysis initiation

^c^adjusted for gender, diabetes, hypertension, HBV, HCV, icodextrin use, APD use, renal Kt/V, albumin and years of dialysis initiation

^d^adjusted for gender, age, alcohol drinking, diabetes, cardiovascular disease, HCV, icodextrin use, APD use, Kt/V, albumin and years of dialysis initiation

^e^adjusted for gender, age, alcohol drinking, diabetes, cardiovascular disease, HCV, icodextrin use, APD use, peritoneal kt/V, albumin and years of dialysis initiation

^f^adjusted for gender, age, smoking, diabetes, hypertension, cardiovascular disease, liver cirrhosis, gout, HCV, icodextrin use, APD use, PET, Kt/V, albumin and Hb

^g^adjusted for sex, age, smoking, diabetes, hypertension, cardiovascular disease, liver cirrhosis, gout, HCV, icodextrin use, APD use, PET, renal Kt/V, albumin and Hb

Table 3 presents the impact of MDPC in patients with and without diabetes. Diabetes patients receiving MDPC had significantly reduced risk of mortality compared to controls with diabetes (adjusted HR = 0.45, 95% CI = 0.25–0.80).

**Table 3:** Hazard ratio of the first episode of peritonitis, technique failure and mortality estimated for multidisciplinary pre-dialysis care group compared to controls by diabetes status

	Patients with multidisciplinary care compared to those without									
	cHR (95% CI)	*p*	aHR (95% CI)	*p*	aHR (95% CI)	*p*	aSHR (95% CI)	*p*	aSHR (95% CI)	*p*
The first episode of peritonitis										
Diabetes										
No	0.82 (0.55,1.22)	0.33	0.95^a^ (0.63,1.45)	0.82	0.97^b^ (0.64,1.49)	0.91	0.96^a^ (0.63,1.46)	0.84	0.98^b^ (0.64,1.50)	0.94
Yes	1.36 (0.80,2.31)	0.26	1.61^a^ (0.90,2.90)	0.11	1.62^b^ (0.90,2.90)	0.11	1.78^a^ (0.97,3.25)	0.06	1.60^b^ (0.89,2.88)	0.11
Technique failure										
Diabetes										
No	0.89 (0.61,1.31)	0.56	0.81^c^(0.52,1.25)	0.34	0.76^d^ (0.50,1.16)	0.21				
Yes	0.78 (0.55,1.09)	0.15	0.92^c^ (0.60,1.41)	0.71	0.96^d^ (0.63,1.45)	0.84				
Mortality										
Diabetes										
No	1.08 (0.61,1.93)	0.78	1.13^e^ (0.55,2.27)	0.74	1.17^f^ (0.58,2.35)	0.66				
Yes	1.30 (0.70,2.42)	0.40	0.42^e^ (0.24,0.75)	0.003	0.45^f^ (0.25,0.80)	0.006				

*cHR* Crude hazard ratio, *aHR* Adjusted hazard ratio, *aSHR* Adjusted sub-distribution hazard ratio, *p P*-value

^a^adjusted for gender, diabetes, hypertension, HBV, HCV, icodextrin use, APD use, kt/V, albumin and years of dialysis initiation

^b^adjusted for gender, diabetes, hypertension, HBV, HCV, icodextrin use, APD use, Renal, albumin and years of dialysis initiation

^c^adjusted for gender, age, alcohol drinking, diabetes, cardiovascular disease, HCV, icodextrin use, APD use, kt/V, albumin and years of dialysis initiation

^d^adjusted for gender, age, alcohol drinking, diabetes, cardiovascular disease, HCV, icodextrin use, APD use, peritoneal Kt/V, albumin and years of dialysis initiation

^e^adjusted for gender, age, smoking, diabetes, hypertension, cardiovascular disease, liver cirrhosis, gout, HCV, icodextrin use, APD use, PET, kt/V, albumin and Hb

^f^adjusted for gender, age, smoking, diabetes, hypertension, cardiovascular disease, liver cirrhosis, gout, HCV, icodextrin use, APD use, PET, renal Kt/V, albumin and Hb

The most common causes of technique failure were death and peritonitis (Table 4), while the most common causes of mortality were cardiovascular disease and infection (Table 5).

**Table 4:** The causes of technique failure

	Multidisciplinary pre-dialysis care	
	No	Yes
	*N* = 126	*N* = 546
No. of technique failure	76	276
Causes		
Death	30 (39.5%)	107 (38.8%)
Peritonitis	22 (28.9%)	68 (24.6%)
Burnout	17 (22.4%)	49 (17.8%)
Medical problems	4 (5.3%)	25 (9.1%)
Inadequate dialysis	3 (3.9%)	16 (5.8%)
Mechanical problems	0 (0.0%)	6 (2.2%)
Exit site or tunnel infection	0 (0.0%)	4 (1.4%)
Encapsulating peritoneal sclerosis	0 (0.0%)	1 (0.4%)

**Table 5:** The causes of mortality

	Multidisciplinary pre-dialysis care	
	No	Yes
	*N* = 126	*N* = 546
No. of mortality	38	133
Causes		
Cardiovascular disease	23 (60.5%)	68 (51.1%)
Infection	7 (18.4%)	44 (33.1%)
Respiratory failure	4 (10.5%)	7 (5.3%)
Cancer	2 (5.3%)	5 (3.8%)
Gastrointestinal bleeding	1 (2.6%)	3 (2.3%)
Accident	0 (0.0%)	3 (2.3%)
Liver failure	1 (2.6%)	1 (0.8%)
Others	0 (0.0%)	2 (1.5%)

## Discussion

Our study demonstrated that the overall risks of developing the first episode of peritonitis, technique failure, and mortality between the MDPC group and the non-MDPC group were not significant. However, diabetic PD patients receiving MDPC had a lower risk of mortality compared to those receiving the usual care.

MDPC for pre-dialysis CKD patients has been shown to be associated with a lower risk of all-cause mortality, a slower estimated glomerular filtration decline, and a decreased risk of progression to ESKD, a lower risk of hospitalization, more planned dialysis starts and a higher proportion of patients initiating dialysis with PD [12, 14, 23, 24]. A retrospective cohort study in the US evaluating 6978 elderly patients with CKD stage 3–5 not yet on dialysis demonstrated that MDPC was associated with a 50% reduction in the risk of death [23]. An open-label, controlled cohort study from Taiwan also revealed that MDPC may decrease the risk of all-cause mortality and reduce the hazard of progression to ESKD for stage 3–5 pre-dialysis CKD patients [14]. Similarly, a recent meta-analysis based on 21 studies also revealed that MDPC reduced the risk of all-cause mortality for patients with stage 4–5 pre-dialysis CKD [12].

The beneficial effects of MDPC might extend to the post-dialysis periods. A small prospective study in Canada including both HD and PD patients revealed that MDPC was associated with a lower risk of deaths after the initiation of dialysis independent of residual renal function, medication use, and laboratory data [9]. A prospective study evaluated the effectiveness of MDPC for patients initiating dialysis at two tertiary care institutions in Vancouver of Canada and in Cremona of Italy [8]. Patients in the MDPC group initiated dialysis at a higher estimated glomerular filtration rate, and had higher hemoglobin, albumin, and calcium compared to those in the non-MDPC group. The non-MDPC group were at an elevated risk of death with a HR of 2.17, compared to the MDPC group. A prospective study from Taiwan found that MDPC was significantly associated with a lower risk of getting the first episode of peritonitis in PD patients [15]. A prospective study in Brazil compared the outcomes between early pre-dialysis care (90 days of follow-up by a nephrology team) and late pre-dialysis care (absent or less than 90 days) in a national cohort of 4107 incident PD patients [13]. The results showed that early pre-dialysis care was associated with better patient survival, but the time to the first episode of peritonitis and technique survival were similar [13]. However, this study failed to adjust residual renal function [13]. In our study, patients in the MDPC group had a higher residual renal function than patients in the non-MDPC group. Thus, patients in the MDPC group were more likely to initiate dialysis earlier than those in the non-MDPC group. In our study, there was no significant difference in laboratory data of albumin, phosphate, hemoglobin, and glycated hemoglobin, distribution of comorbidities, and duration of break-in period between the two groups. In a model without adjustment for residual renal function, MDPC was associated with a lower risk of mortality. However, there was no significant difference between the two groups in risks of mortality after adjustment for residual renal function. The adjustment of residual renal function could reduce the lead-time bias.

Diabetes is a major risk factor for peritonitis, technique failure, and mortality in PD patients [7, 25]. In other words, PD patients with diabetic have a worse prognosis than those without diabetes. There was no difference in the care of MDPC program between diabetic and non-diabetic patients. In our study, the subgroup analysis demonstrated that PD patients with diabetes under the care of MDPC program had a much lower risk of mortality than those in the non-MDPC group.

Although care of PD patients after dialysis initiation are also multidisciplinary approach with involvement of nephrologists, dietitians, and nurses, there might be a legacy effect of MDPC. The positive effects of MDPC include selecting healthier PD candidate, adaptation of positive attitude toward illness, enablement of self-care technique, improvement in patient compliance with treatment, maintenance of a healthier lifestyle, timely initiation of renal replacement therapy, and greater understanding of PD complications. The possible reasons why the diabetic PD patients might get more benefits through this program included better residual renal function preservation, better glycemic control, better blood pressure control, etc. [26]

The strength of this study is the use of a well-organized database of medical records collected in a recent decade with the sample size large enough to evaluate outcomes after a long follow-up period. There are limitations in this study. This study was observational and retrospective in design. Medications such as angiotensin-converting enzyme inhibitors and angiotensin receptor blockers were not included in the analysis. The majority of the study population were less than 70 years. Thus, we did not further analyze the female younger subgroup. In addition, the assignment of MDPC was up to the preference of physicians and patients. There might be a selection bias. However, multivariate analyses were preformed to reduce the bias. The observational study contains valuable information that could be conveyed to the nephrology community.

In conclusion, patients in the MDPC group were more likely to initiate dialysis earlier than those in the non-MDPC group. There were no significant differences in time to the first episode peritonitis, and risks of technique failure and mortality between the MDPC group and the non-MDPC group. The MDPC program could reduce the risk of death for patients with diabetes, compared to those under the usual care.

## Data Availability

The data that support the findings of this study are available from the corresponding author upon reasonable request.
